# Consent in the dark: Can living kidney donation proceed amid marital infidelity?

**DOI:** 10.3389/ti.2026.16580

**Published:** 2026-10-02

**Authors:** Crystal Lim, Teck Chuan Voo, Peter Chiu-Leung Chow, Lalit Kumar Radha Krishna

**Affiliations:** 1 Singapore General Hospital, Singapore, Singapore; 2 SingHealth Duke-NUS Medical Humanities Institute, Singapore, Singapore; 3 Changi General Hospital, Singapore, Singapore; 4 National Cancer Centre Singapore, Singapore, Singapore

**Keywords:** informed consent, living kidney donation, marital infidelity, program integrity, public trust

## Abstract

Living kidney donation requires rigorous medical, psychosocial, and ethical evaluation to safeguard donor welfare, ensure valid informed consent, and prevent wrongful exploitation. In Singapore, the Human Organ Transplant Act mandates review by a transplant ethics committee (TEC) for all living-donor transplants. However, ethical judgment is not reducible to TEC approval or procedural compliance; transplant programs themselves carry a broader responsibility to evaluate relational dynamics that may affect voluntariness and authenticity of consent. This paper examines three cases involving concealed marital infidelity history in either kidney donor or recipient candidates or ongoing extramarital relationships in potential kidney donors and recipients that raised ethical concerns. Key issues include the materiality of information for informed consent, risks of psychosocial harm, integrity of caregiving arrangements, and potential institutional complicity in ongoing deception. While personal moral discomfort alone does not justify rejection, undisclosed relational complexities may warrant conditional deferral to protect donor welfare, program credibility, and public trust. In certain contexts, proceeding without disclosure may be ethically permissible when consent remains autonomous, donor privacy is respected, and anticipated harm is minimal. Transplant programs must balance substantial clinical benefit with ethical stewardship to preserve public confidence and the moral credibility of living donation.

## Graphical Abstract

**Figure:**
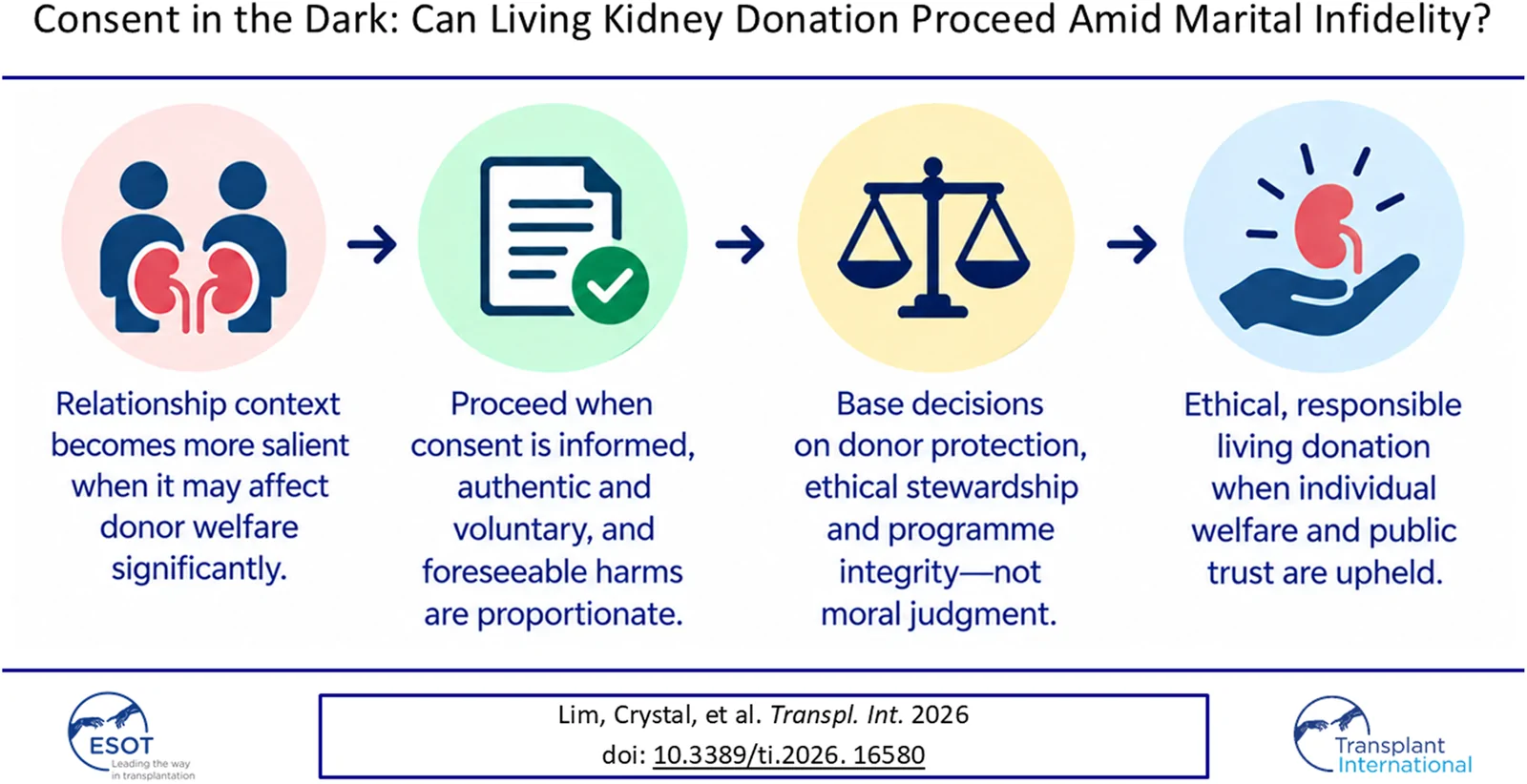

## Introduction

In Singapore, the Human Organ Transplant Act (HOTA) mandates transplant teams to conduct medical and psychosocial evaluations of living-donor candidates and obtain unanimous approval by a national Transplant Ethics Committee (TEC) [1]. Each TEC meeting comprises of a layperson and two doctors uninvolved in transplant medicine: one from the transplant hospital and another from the community. They are drawn from a national TEC list overseen by the National Organ Transplant Unit. Recipient and donor candidates are interviewed separately by the TEC, who also rely on detailed medical and psychosocial reports in their ethics review.

Ethical scrutiny by TECs focuses on three considerations: donor welfare, valid informed consent, and prevention of harmful or exploitative payment or trading. A case rejected by a TEC cannot seek transplant elsewhere in Singapore. Transplant teams must therefore ensure cases submitted for TEC review have sufficient ethical merit [1].

This paper examines three cases in which transplant candidates—between 40- and 60-years-old—voluntarily disclosed previously concealed intimate relationships during pre-transplant evaluation. Although the disclosures were not medically relevant, they raised difficult ethical questions about donor consent, recipient privacy, and the responsibilities of transplant teams when relational information could materially affect the decision to donate or receive a kidney.

## Case series

### Case A: recipient infidelity

Case A revolves around donor candidate (D_a_) who wished to donate a kidney to emancipate the spouse (R_a_) from life-long dialysis and preserve the marriage and family unit. R_a_ voluntarily disclosed to the transplant team information about an extra-marital relationship lasting nearly a decade and that it had recently ended. R_a_ refused to disclose this fact to D_a_.

### Transplant team’s deliberations

Awareness of R_a_’s extramarital relationship called into question the team’s ability to abide by the Consensus Statement on the Live Organ Donor that requires both parties to provide valid informed consent [2]. D_a_’s donation offer was made on the presumption of mutual marital fidelity and shared commitment to the marriage. Information about R_a_’s infidelity was arguably material to D_a_’s deliberations as D_a_ might not donate a kidney upon being aware of R_a_’s infidelity. There was concern about foreseeable psychosocial harm [3, 4] to D_a_ should the marriage implode [5, 6] because of these factors.

This ethical conflict may be framed as D_a_’s right to material information versus R_a_’s right to privacy, particularly when the information was shared in good faith. One could argue that R_a_’s disclosure represents a recommitment to the marriage. However, the recency of this ending of the affair itself is ethically significant. Might R_a_ feel coerced to end the affair simply to secure the transplant? Might it be the product of implicit coercion given D_a_’s desire to maintain the marriage and family unit through the sacrificial bodily gift? Was R_a_’s decision to recommit to the marriage made amidst psycho-emotional instability, emotional volatility, and ethical or moral duress given D_a_’s altruistic gesture? Informed consent in such a circumstance would also be called into question.

Information about R_a_’s extramarital relationship did, however, raise the concern that D_a_ might potentially use his donation to pressure R_a_ to reconciliation should R_a_’s infidelity became known [7–9].

### The decision

The team has duties to safeguard its reputation and the ethical integrity of its decision [10]. Some members felt that allowing this transplant would breach this responsibility because the institution would be complicit. Dissonance amongst the team led to a majority decision to defer but not prohibit the transplant. In theory it would provide R_a_ with a chance for grief and reflection or to undergo counselling to sort out emotional needs and relational priorities. However, some members perceived this decision as exerting undue pressure on R_a_ to “come clean” by the time of the next review.

Before coming to terms with the outcome, R_a_ was upset that transplant deferral consequent to candid and voluntary disclosure of information led to personal disadvantage and felt penalized for honesty.

### Case B: donor infidelity

In this scenario, the roles were reversed. A donor candidate (D_b_) wished to donate to the spouse (R_b_) to alleviate R_b_’s suffering. D_b_ voluntarily disclosed to the transplant team about a three-year extra-marital affair preceding R_b_’s dialysis-dependency. R_b_’s fortitude and unyielding care for the family amidst illness hardship made D_b_ determined to become a good spouse by ending the affair. D_b_ then waited a year to allow temporal and emotional space before broaching kidney donation to R_2_, who gratefully accepted the offer.

### Transplant team’s deliberations

This case was dissimilar to Case A. Here, ethical concerns centered primarily on the recipient instead of the donor.

At the fore are concerns that omission of D_b_’s marital infidelity facilitates R_b_’s consent for transplant through ignorance, threatening R_b_ autonomy. R_b_ might feel repulsed at the idea of having D_b_’s kidney if in knowledge of D_b_’s infidelity. On the other hand, while emotionally significant, this information was not clearly material to R_b_’s evaluation of transplant risks and benefits. R_b_’s reason for accepting the kidney—to escape the burdens of dialysis—was independent of D_b_’s marital infidelity. Concealment of D_b_’s prior infidelity would not alter R_b_’s surgical risk, transplant prognosis, or expected clinical outcome. R_b_’s quality of life and that of the family’s would improve with a transplant, which are consistent with R_b_’s goals. By contrast, D_b_’s past infidelity is ethically relevant insofar as disclosure might cause significant relational harm [4, 5, 11, 12], jeopardize the transplant, and thereby indirectly worsen R_b_’s medical situation.

On the other hand, we should not assume that R_b_ would want to know about D_b_’s marital infidelity. R_b_ might want to exercise negative autonomy and refuse relevant information on spousal fidelity. The preference to know about spousal infidelity varies across individuals [12] and it would not be possible to ascertain R_b_’s preference without revealing the truth. There is concern that R_b_ might feel coerced to forgive D_b_ and even stay within the marriage as a result of knowing [12, 13]. Proceeding with the transplant raises ethical concerns because of resultant harm from concealed relational truths.

Some members challenged that D_b_’s admission of regret and some sense of guilt compelled the donation offer. While D_b_ acknowledged that the donation would represent an act of contrition [14], D_b_’s motivation was R_b_-centered—improving R_b_’s quality of life and lifespan—not compelled by remorse or redemptive need. This reflected D_b_’s personal choice in moral responsibility-taking, an expression of autonomy, and is ethically supportable.

Further, D_b_’s ending the extramarital relationship for a year represented a recommitment to the marriage. The donation need not trap or prevent D_b_ from leaving the marriage should the infidelity come to light. The factors that led D_b_ to embark on the extramarital relationship may resurface and the donation will not inhibit D_b_ from exercising personal choices.

Beyond the duty to ensure recipient autonomy, the transplant team has a duty to safeguard the team’s reputation and the ethical integrity of the decision-making process [1, 10]. Even if the medical outcome is beneficial, ignorance of morally salient relational truths raises concerns about respect for individuals.

Similar arguments to the previous case as to the significance of knowingly omitting the information also raised concerns about ethical, professional, and institutional integrity.

### The decision

The transplant assessment team was divided in their perspectives. Overlooking morally salient facts that could reasonably affect recipient decision-making could manifest ethical compromise. However, there was no donor deception or coercion in this case, and the donation would imperatively fulfil R_b_’s wish for a transplant. Deferring the transplant would not rewrite the marital history but could instead delay R_b_’s access to improved quality of life, potentially causing greater harm. In balancing these considerations, our team recommended that proceeding with the transplant was deemed the lesser of two harms.

D_b_ donated the kidney and admittedly did not make any subsequent disclosure of his marital infidelity history to R_b_.

### Case C: recipient and donor infidelity

In the third scenario, the donor (D_c_) and the recipient (R_c_) concealed their intimacy from their spouses and presented themselves as close friends. D_c_ maintained that the donation offer was motivated by altruism, friendship, and compassion, and R_c_ sought the transplant for dialysis emancipation.

### Transplant team’s deliberations

In principle, the transplant could be supported because the threshold for informed consent was met. Both parties comprehended and accepted the risks and benefits of the transplant and treatment alternatives for kidney failure and were voluntary in their decisions [15, 16]. Importantly, informed consent was owed to the donor and the recipient—not to their spouses—even if the spouses were misinformed about the nature of their relationship.

On the surface D_c_’s willingness to donate seemed informed and free of coercion. Doubts were cast, however, given the illicit nature of the relationship. There were concerns that D_c_’s offer could be coerced as “proof” of devotion, fear of abandonment, and the promise of relationship continuity in reciprocity for donation. There were also questions over the relationship power dynamics that favored R_c._

Though not as evident in the discussions with R_c_, questions were also raised as to whether R_c_ would feel compelled to stay in this relationship because of this donation.

It was clear that D_c_ and R_c_ had rights to privacy, and the healthcare team lacked justification to breach confidentiality. This then placed the team in a bind. The transplant would be contingent on continued deception of D_c_ and R_c_’s spouses. Would the team not be complicit in this? What were the team’s liabilities beyond the moral and ethical for any risk or disadvantage to D_c_ and R_c_’s spouses should transplant complications befall D_c_ and R_c_? Additionally, when spouses are misled about the nature of the relationship, post-operative support will be built on false premises, placing spouses in caregiving roles they may not have agreed to. This raised fairness concerns for the spouses.

While the risk of exposure of the extra-marital relationship might not be high, the risk to the publicly accountable team is not insignificant. There will be narrative harm to institutional stakeholders from supporting this transplant where scrutiny will be on institutional complicity in misleading the spouses and their families, instead of how the case is technically justified on grounds of informed consent. The situation is further compounded if marital breakdown or family conflict involving children occur [17, 18].

### The decision

The team reached a unanimous decision on conditional deferral for this case. Most team members wanted to uphold socio-moral norms and protect institutional integrity. They were also concerned that emotional entanglement and concealed deception warranted pause.

Deferral permits time for potentially remediable action, such as D_c_ and R_c_ deciding on the way forward for their relationship: whether to end it, end their marriages, or disclose their affair to their spouses. Decision on whether they want to further pursue living-donor transplant can then follow. While deferral might be interpreted by some as interference, the team believed that there appeared to be no better alternatives that could address the ethical concerns.

While disappointed with the outcome—particularly R_c_—both accepted it and understood the transplant team’s perspective.

## Discussion

Although the disclosures in these cases involved different relational circumstances, they raised a common ethical question: should living-donor transplantation proceed when concealed intimate relationships materially affect the relational context in which donation occurs? These cases therefore invite closer examination of how relational knowledge interacts with informed consent and the ethical responsibilities of transplant programs.

### Donor vulnerability and integrity of consent

It is not the transplant team’s role to demand disclosure when information suggesting concealed infidelity involving the donor, recipient, or both comes to light. Rather, the team must determine whether such information could affect the validity of consent by either party.

Spousal living donations are typically motivated by love, devotion, marital commitment, a desire to improve the spouse’s quality of life, and preservation of the family unit [19]. A decision to donate based on a material misunderstanding of the true nature of the donor–recipient relationship is ethically significant because it compromises autonomous decision-making [16].

In living-donor transplantation, the donor undergoes surgery and assumes the risks of bodily harm without direct medical benefit, while the principal medical benefits are accrued to the recipient [8, 19]. This asymmetry of risk heightens the ethical importance of donor consent. Consequently, factors that undermine the donor’s ability to provide informed consent should carry greater ethical weight than those affecting the recipient, as illustrated in Case A.

The ethical legitimacy of living-donor transplantation rests on protecting donor welfare and ensuring the authenticity of the donor’s voluntary choice [1]. When concealed intimate relationships materially shape the relational context in which donation occurs, transplant teams must consider whether the donor’s decision rests on a material misunderstanding. Respect for privacy remains important, but it does not absolve transplant programs of their responsibility to safeguard the integrity of donor consent.

### Relational context and impact

In spousal kidney donation, the meaning of accepting a bodily gift is deeply relational. Such donations are commonly grounded in values of commitment, fidelity, shared suffering, mutual sacrifice, and preservation of the family unit [8, 19, 20]. An extra-marital relationship destabilizes these relational foundations.

Although it is not the role of the transplant team to adjudicate marital conflict or compel disclosure, the team must consider foreseeable relational and psychosocial harms that could arise from concealed intimate relationships in spousal donation. Recent relational rupture is known to introduce instability, emotional volatility, and unresolved conflict, with repercussions for the marriage and family [4–7, 11, 12].

Empirical literature suggests that preferences regarding disclosure of spousal infidelity are heterogeneous. Some individuals prefer to know, while others deliberately avoid such knowledge as a coping strategy [8]. In practice, however, these preferences cannot be determined without revealing the concealed relationship.

If an affair is later discovered or disclosed—whether intentionally or inadvertently—the recipient would likely experience profound betrayal and distress [3, 4, 7]. The transplanted organ may become a lasting symbolic reminder of relational deception, with surgical scars embodying an irrevocable bodily gift received under conditions of incomplete relational understanding. The donor could also experience significant harm and the surgical scars will be a permanent reminder of an irreversible bodily sacrifice made amid marital deception.

When the anticipated benefit to the recipient is substantial and donor risk acceptable, principles of beneficence and proportionality favor proceeding, particularly in the absence of evidence suggesting imminent marital breakdown or foreseeable post-donation harm.

### Deferral as a proportionate response

In some cases, conditional deferral—rather than outright prohibition—offers a proportionate response. Deferral provides time for the donor and recipient to address relational instability, consider disclosure where appropriate, and ensure reliable post-operative support—factors directly relevant to donor welfare, voluntariness of consent, and program integrity.

Since these cases did not involve an urgent need for kidney transplant and given Singapore’s legislation where a rejection effectively precludes transplantation anywhere in the country, deferral preserves the possibility of future transplantation by allowing the donor-recipient pair to be reconsidered once the identified prohibitive factors have been adequately addressed or resolved.

Deferral therefore preserves donor autonomy while safeguarding clinical prudence, institutional credibility, and public trust. Importantly, it represents proportional restraint rather than moral adjudication of private wrongdoing.

### Transplant team’s duties and program integrity

Beyond protecting donor welfare, transplant programs and TEC members are statutorily required to consider “public interest and community values” when evaluating applications for living-donor transplantation, according to Human Organ Transplant Regulations 2004 [1]. Although these concepts are intentionally broad and undefined, they must be interpreted in context.

Public interest generally refers to society’s collective welfare, rights, and institutional protections. Community values refer to socio-moral norms that shape expectations about how individuals ought to treat one another, including within intimate relationships [21]. These considerations may reasonably extend to preventing foreseeable harm to spouses, families, and marital relationships.

Institutional integrity is therefore central to ethical transplantation practice. Approving a donation in the context of known relational deception risks reputational harm and erosion of ethical credibility. Such cases may create the impression that living donation is insufficiently regulated or ethically compromised, potentially weakening public confidence in psychosocial and ethical oversight.

Transplant programs must therefore balance clinical benefit with broader ethical responsibilities: protecting donor welfare, ensuring meaningful consent, safeguarding participants from exploitation or foreseeable harm, and maintaining living donation as a transparent and ethically defensible practice.

The Declaration of Istanbul [22] emphasizes that governments and transplant programs must develop ethically sound transplant systems [23]. Ethical soundness includes safeguarding public trust, maintaining institutional integrity, and ensuring that living donation remains grounded in principled donor protection [1, 10]. A favorable recipient outcome cannot justify exposing a donor to undue risk or ethical compromise.

## Conclusion

Transplant programs should not reject living kidney donation solely on the basis of moral discomfort with aspects of the donor–recipient relationship. However, confidentiality and respect for autonomy alone are insufficient to justify proceeding when transplant teams become aware of concealed extramarital relationships that materially affect the relational context of donation.

Ignoring such relational realities risks reducing ethical oversight to procedural compliance rather than principled judgment consistent with the team’s duty of stewardship. Transplant programs must therefore consider not only medical information but also relationally material information that could reasonably influence a donor’s decision to proceed.

Ethically sound transplantation requires governance beyond formal TEC review. Transplant teams themselves play a critical role in ethical stewardship—ensuring rigorous psychosocial evaluation, assessing the authenticity of consent, and determining whether cases should proceed to TEC review or be deferred to address underlying ethical concerns^20^.

To the best of our knowledge, this is the only known work discussing the impact of concealed intimate relationships in living-donor transplant. The paucity of literature suggests an important gap in transplant ethics scholarship. Further empirical and normative work is needed to guide transplant programs confronting these ethically complex situations.

## Data Availability

The raw data supporting the conclusions of this article will be made available by the authors, without undue reservation.
